# Variation in NHS utilisation of vault smear tests in women post-hysterectomy: A study, using routinely collected datasets

**DOI:** 10.1186/1472-6874-8-6

**Published:** 2008-03-28

**Authors:** Helen J Stokes-Lampard, John Macleod, Sue Wilson

**Affiliations:** 1 Department of Primary Care and General Practice, University of Birmingham, Edgbaston, Birmingham, West Midlands, B15 2TT, UK; 2 Department of Social Medicine, University of Bristol, Canynge Hall, Whiteladies Road, Bristol, Avon, BS8 2PR, UK

## Abstract

**Background:**

20% of women living in the UK have a hysterectomy during their lifetime, levels are higher in the USA, making it one of the most commonly performed major surgical procedures. Understanding of the indications for hysterectomy and of the rationale for follow-up of women post hysterectomy is currently limited. Guidelines concerning follow-up by means of vaginal vault cytology tests exist but these are not based on 'gold standard' evidence. Furthermore, the extent to which current practice reflects these guidelines is unclear. This study aims to determine the factors associated with variability in hysterectomy rates and subsequent follow-up after surgery by use of the vaginal vault smear cytology test.

**Methods/Design:**

All women resident in the West Midlands region, of the United Kingdom, who had a hysterectomy operation between 1st April 2002 and 30th March 2003 will be identified from the Hospital Episodes Statistics database which also contains proxy data on deprivation status, derived from postcode and self declared ethnicity. These data will be linked to regional cervical screening records for each woman and histopathology laboratory records from the relevant hospitals. Study objectives are to describe: Indications for the hysterectomy operation, histology at hysterectomy, subsequent follow-up by use or non-use of vaginal vault cytology tests and variation between histological groups. Additionally the data will be categorised according to a woman's cytology screening history prior to surgery (i.e. always normal, borderline, resolved abnormalities, CIN etc) and these different groups compared. Variations in these outcomes according to age, deprivation and ethnic group will also be examined. Analysis will be undertaken using SPSS.

**Discussion:**

This study will clarify patterns of current practice in one large English region and determine whether this practice reflects existing guidelines. The study will also strengthen the evidence base for future guidelines.

**Study registration:**

National Research Register N0138173331

## Background

Surgical removal of the uterus (womb) is a 'hysterectomy' operation; over 98% women who have their uterus removed also have the cervix uteri or 'neck' of the uterus removed at the same time, a total hysterectomy[1]. This leaves the vagina as a pouch with a blind end at the site of amputation of the cervix. There were around 39,000 hysterectomy procedures undertaken in the UK in 2005 [2], a cumulative lifetime incidence of 20% [3,4], making it one of the most frequently performed major surgical procedures [5].

The most common indication for a hysterectomy is menorrhagia which accounts for 46% of all hysterectomies. Prolapse accounts for a further 20%, and fibroids (or leiomyoma) another 18%–21% [6]; 90% of hysterectomies are performed for benign or noncancerous conditions [7].

Hysterectomy is inversely associated with social class and education with women from lower social classes being more likely to have a hysterectomy [3]. Several large cohort studies have examined the indications for hysterectomy in the UK, however none has published details of how these women are followed-up or the cost-effectiveness of the vaginal vault cytology test (known colloquially as a 'vault smear') in the population [3,4,6]. A vault smear is a cytological sample taken from the blind end of the vagina; the vault smear is used a means of identifying recurrent cervical cancer or the development of vaginal neoplasia. Total hysterectomy is usually a reason for ceasing recall from the routine cervical screening programme, as the cervix is no longer present.

No international consensus exists on the appropriate extent of cytological screening in women who have undergone hysterectomy, as evidence for the appropriate use of vaginal vault smears post-hysterectomy and the optimum period of follow-up is sparse. The majority of published studies recommend the use of vaginal vault smears in the follow-up of women who have had a hysterectomy subsequent to the diagnosis of an invasive tumour of the cervix uteri, or where invasive disease is an incidental finding at hysterectomy [8-12]. A systematic review of the literature [13] could identify no robust controlled trials that establish the value of the follow-up, by vault smears, for women who have had a hysterectomy for benign indications. The available evidence does, however, suggest that the vault smear test has a very low positive predictive value when used as a screening tool in the absence of symptoms or clinical signs [8,9,14]. Therefore, most commentators recommend that vault smears for post-hysterectomy follow-up are only required for women who have had a histological diagnosis of Cervical Intraepithelial Neoplasia (CIN) III or frank malignancy [10-12]. Some go as far as to say that even in women with previous cytological abnormality, vault smears should be limited to those who present with symptoms or in whom an abnormality is detected clinically [15].

Currently the UK National Health Service Cervical Screening Programme (NHSCSP) guidelines for the use of vaginal vault smears are: [16,17]

(i) for women on routine recall for at least 10 years prior to hysterectomy, and no CIN in the histopathology sample at hysterectomy, no vault cytology is required.

(ii) for women with less than 10 years' routine recall and no CIN at hysterectomy, a sample should be taken from the vaginal vault six months after surgery and there should be no further cytology follow-up if it is negative.

(iii) for women with completely excised CIN at hysterectomy, a sample should be taken from the vault at 6 and 18 months after surgery and there should be no further cytology follow-up if both samples are negative.

(iii) for women with incomplete or uncertain excision of CIN follow-up should be conducted as if the cervix were still in situ (i.e as for low and high risk follow-up).

(iv) women who have undergone a hysterectomy but where the cervix is not completely excised are treated as if the cervix were still present and as such they remain in the normal cervical screening programme.

In a survey of primary healthcare professionals in South Birmingham, UK, half of all vault smear tests were conducted in the primary care setting but primary healthcare professionals' knowledge about the role of the test was poor [18]. Professionals whose knowledge about the test was best, performed the test least often, and only one significant abnormality was detected in over 5,000 vault smear specimens [18]. However, there is no recent reliable data on the patterns and variability in vault smear follow up and the extent of compliance with the national guidelines is unknown.

No large-scale population studies have been undertaken to establish the actual patterns of follow-up, by use of vault smear tests, after hysterectomy. One American study compared the results of vault smears with cervical smears, in matched controls, and noted that there was a significantly reduced risk of test abnormality in those followed up post-hysterectomy [19]. Vault smear tests undertaken in asymptomatic women can have a high false positive rate thus reducing the usefulness of the test [8]. Large-scale national cohort studies have reported on the socioeconomic distribution of patients undergoing hysterectomy [4] and the distribution of hysterectomy type (vaginal versus abdominal) [6], but none has examined the socioeconomic distribution of the histology results at surgery and the socioeconomic distribution of any subsequent follow-up by means of vault cytology.

No international consensus exists as to the most appropriate follow up by vault smear test after hysterectomy [18]; the UK guidelines are not based upon 'gold-standard' evidence [16,17]. With the increasing pressure on diagnostic and treatment services, the evaluation of diagnostic services and identification of inappropriate testing are necessary prerequisites to improving the efficiency of service provision. Thus there is a need for an adequately powered, population based study to consider the issue of hysterectomy follow up, the outcome of which may be used to inform national guidelines and encourage the teaching of best practice.

### Key objectives

- Estimate age and socioeconomic specific incidence rates for hysterectomy, in the West Midlands.

- Describe indications for hysterectomy in West Midlands.

- Describe variations in incidence and establish those factors associated with variability.

- Establish the current pattern of follow-up after hysterectomy by means of vaginal vault cytology test.

## Methods/Design

### Study design

**Figure 1:**
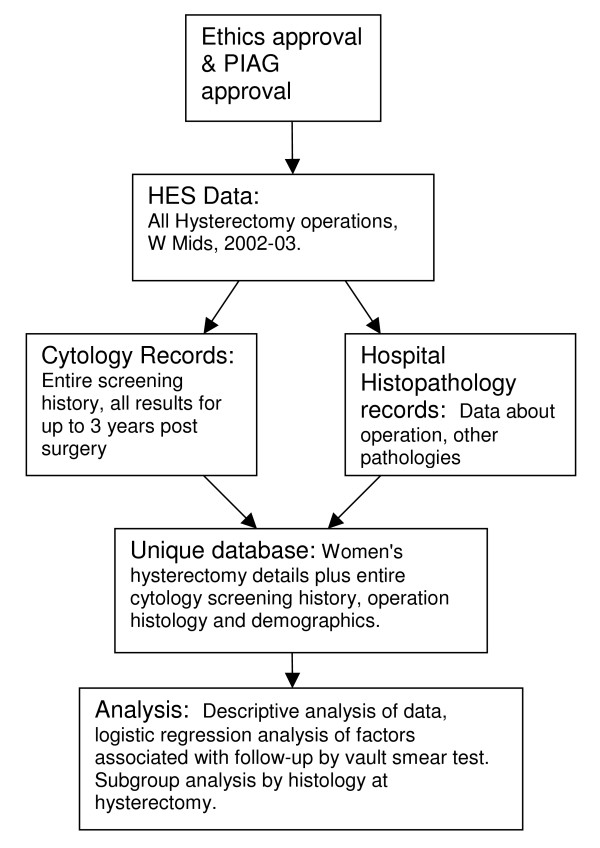
Study design.

This retrospective population-based analysis of routinely collected data will link data, from three key sources and hence enable us to address the study objectives. Figure 1 illustrates the main stages of the study.

Hospital episode statistics (HES) is the national statistical data warehouse for England of the care provided by NHS hospitals and for NHS hospital patients treated elsewhere. It is the data source for a wide range of healthcare analysis for the NHS, Government and many other organisations and individuals. For this study HES will be used to identify the population of women having a hysterectomy during the 2002 – 2003 fiscal year (1 April 2002 – 30 March 2003). These women's cervical screening history records will be obtained from the local health authority cytology databases; complete records of cytology results for all women who have had cervical screening within a local catchment area, ten such areas cover the West Midlands Health Authority region. All these cytology databases use the same suite of software and contribute to national statistics to ensure uniform reporting. These data will be linked and pseudo-anonymised to just NHS number: A national, unique, ten-digit identifier that makes it possible to share patient information across the whole of the NHS) then supplemental data about specific diagnosis and laboratory advice will be added from hospital pathology laboratory records [20]. The dataset will be fully anonymised to ensure patient confidentiality.

### Selection and exclusion criteria

The cohort will include all women resident in the West Midlands region who had a hysterectomy operation during the defined study period.

### Population

The West Midlands conurbation of 5.2 million inhabitants covers a very diverse population: It includes urban, suburban and rural areas ranging from some of the most to the least affluent wards in the country [21]. This represents a 10% sample of the population of England and Wales, (11% of England) and has, on average, a similar age, sex and socioeconomic profile to the UK as a whole [22].

Black and minority ethnic groups make up 11.3% of the West Midlands population, however the range within the region is quite striking with the 'Birmingham' Local Health Authority recording 29.6% of persons classifying themselves as being of black or minority ethnic group (second only to London) but the 'Staffordshire Moorlands' Health Authority figure is only 0.7% [22].

### Study methodology

The study depends upon the extraction of data, from three separate routinely collected datasets, which will then be combined into a relational database (Microsoft Access 2007) in a format suitable for analysis and fully anonymised. Table 1 summarises all the data items to be requested and those that will be used to facilitate data linkage between the three databases.

**Table 1:** Summary of extracted data and derived data items to be obtained from the three sources.

	**Hospital Episode Statistics (HES)**	**'Exeter' Cervical Screening data**	**Local Hospital Histopathology data**
**Identifiers for linkage**	NHS Number, date of birth, postcode of home address.	NHS Number, date of birth, postcode of home address.	NHS Number.
**Data items**	Ethnicity, dates of hysterectomy operation, admission and discharge, surgical operation code(s) OPCS, diagnosis code(s) SNOMED morphology, hospital, consultant, GP and PCT of residence.	Dates of all smear tests, results of those smear tests and recommended follow up, details of smear taker, registered GP, current cervical screening status and date of death (where applicable).	Details of histology at hysterectomy, details of all specimens removed, details of results of analysis (morphology or diagnosis codes), full details of smear tests processed at the laboratory.
**Data to be thus derived or calculated**	Deprivation score (IMD), duration of stay, diagnostic grading of operation (benign vs pre-malignant or malignant disease), age at surgery.	Deprivation score (IMD), scoring of screening history using algorithm, whether any vault smears undertaken (also using operation date from HES).	Confirmation of whether total or subtotal hysterectomy undertaken.
**Explanatory Notes**	Request full data on all West Midlands resident women who had a hysterectomy during the year, 1^st ^April 2002 to 30^th ^March 2003. Requires approval of HES security and confidentiality advisory group.	Request full cervical screening histories on all women identified in HES database. Requires individual permission from each of 10 database controllers.	Request full histopathology records on all women with NHS numbers identified in HES. Requires permission from each of 17 hospitals ethics committees and heads of department.

### Data sources

The Hospital Episode Statistics (HES) dataset is managed by Northgate Information Solutions and access to sensitive confidential information requires permission to be granted by their security and confidentiality advisory group (SCAG) [23]. It is from this database that hospital in-patient data will be extracted: Data about all hysterectomy operations will be requested and then categorised as 'total' and 'sub-total' hysterectomies with the sub-total operations being excluded after verification.

The NHS Information Authority (NHSIA) has control over the Exeter database: The NHSIA is a 'special health authority' formed in 1999, the 'Exeter' system or now, more correctly the 'National Health Applications and Infrastructure Service' (NHAIS) is an integrated suite of software used by all health authorities at a regional level for holding administrative details of patients on GPs lists and used to manage patient registration, GP payments, breast and cervical screening programme [24]. The NHSIA will provide the following dataset: All women, residing in the West Midlands region and identified from the HES dataset, who have ever had a smear test (cervical or vaginal).

Currently there are 17 cytopathology units in the West Midlands region and over 20 units where hysterectomy operations are performed routinely. To obtain accurate data concerning the histology at hysterectomy, it will be necessary to access the histopathology records from some hospitals directly. Since 1995 all histopathology laboratories in the West Midlands have had stand alone, computerised databases of their clinical records and thus data extraction will not require access to patient case notes, just to the electronic histopathology and cytopathology records.

Once matching and merging of all three data sets is complete, all data will be anonymised to the level of unique study identifier, age at hysterectomy and deprivation index; thus NHS number, surname, date of birth and postcode will be replaced with less identifiable indices.

The whole project was registered on the National Research Register in December 2004 entry: N0138173331. The approvals of both Multi Research Ethics Committee (MREC) and Patient Information Advisory Group (PIAG), have already been granted (MREC – West Mids MREC Approval granted on 27th April 2005 Ref: 05/MRE07/27, PIAG Full approval – 7th March 2006 Ref: 4-05(e)/2005). SCAG of HES granted approval for access to their data on 11th April 2006, Ref: ET0693.

### Ethical issues

This project justifies the use of confidential patient data because the likely benefits to society outweigh the implications of that transient loss of confidentiality. The outcome of this research may have significant implications for the general population of women thus it may be argued that it is in the public's best interests for the research to be undertaken. There is no intention to feed information back to the individuals involved or take any decision that affects them. There are no practicable alternatives to access patient data of this quality that would be of equal effectiveness. Confidentiality has been planned from the outset of this study, and data will be anonymised as soon as is practicable after data validation has taken place.

### Power calculation/justification of sample size

This is a pragmatic sample of all women in the West Midlands Region who underwent a hysterectomy operation but sample size calculations were undertaken during protocol development to ensure the study will have sufficient power to detect important differences.

The key groups of women are specified by their histology result at the time of surgery (benign/cervical intraepithelial neoplasia (CIN)/malignant) as this determines their recommended follow up according to national guidelines. The data in Table 2 were used to provide the estimated proportions on the basis of previous research by the authors (Stokes-Lampard H, Wilson S, Waddell C, Bentley L, Vaginal vault smears: 10-years of data from a tertiary centre (Birmingham Women's Hospital NHS Trust), awaiting publication.)

**Table 2:** Estimated numbers of women in each histology category

	Sub-Total* (Excluded)	**Benign**	**CIN**	All Cancers	**Totals**
**Not followed up VS**	*N*/*A*	3240	90	23	3,353
		90%	20%	10%	
**Followed-up VS**	*N*/*A*	360	360	202	922
		10%	80%	90%	
**Totals**	225	3600	450	225	**4,275**
	*5%*	80%	10%	5%	(95%)*

* with 5% excluded for being sub-total operations.

### Assumptions

▪ 4,500 hysterectomies annually in West Midlands.

▪ 80% of hysterectomies undertaken for benign indications

▪ 10% for CIN

▪ 5% for cancer

▪ 5% will sub-total hysterectomies and excluded from analysis (approximately 225 cases excluded).

For the benign histology group, a sample of 1,800 women will be sufficient to estimate prevalence of follow up to within +/-1% (95% CI); for the CIN and cancer groups the estimate would be to within +/-4% (95%CI). Thus the expected sample should be more than adequate and does not need to be expanded further.

### Analysis

Will be undertaken using SPSS for Windows statistical software, with queries in Microsoft Access 2003^® ^used to produce descriptive data. A substantial amount of descriptive analysis of the data will be undertaken i.e. frequency distributions, cross tabulations and simple proportions, much of which will be amenable to visual formats.

Comparison of the whole study population with the West Midlands and England populations (2001 census data) with respect to Index of Multiple Deprivation [22], age, and ethnicity will be undertaken initially and the results tabulated. Then age-specific incidence rates (5-year age bands) for hysterectomy will be calculated.

Pre specified analysis will be used to describe and explore those factors potentially affecting variability within the three main research areas i.e. i. Indications for hysterectomy, ii. duration of hospital stay after hysterectomy and iii. follow up after hysterectomy by means of the vaginal vault smear cytology (vault smear test).

Hospital diagnosis (obtained from HES records) will be used as a proxy for indication for hysterectomy. Indications for hysterectomy will be explored with respect to deprivation score, age, ethnicity and hospital where surgery took place. Duration of hospital stay will be described and explored with respect to age, ethnicity and deprivation score. Each of these will be considered further by histology type; benign, CIN (further subdivided into CIN I, II & III) and malignant. Pre-hysterectomy cervical cytology data will be explored with respect to age, deprivation and ethnicity. This data will be coded according to 'per protocol' and 'non-protocol' patterns, according to UK guidelines, using an algorithm constructed by the cancer registry in the West Midlands, this is to ensure standardised results to facilitate future analysis. Post-hysterectomy vault cytology will be explored with respect to age, histology type, deprivation and ethnicity. Most of this descriptive analysis will be explored using χ^2 ^and t-tests as appropriate.

Multiple regression analyses will aim to determine the relative importance of those factors (i.e. age, deprivation index, hospital of procedure and ethnicity) on the outcomes of interest.

Finally, differences in the rates of vault smear cytology between the different histology sub-groups will be further explored using Kaplan-Meir survival analysis. The sub-groups will be compared with national guidelines to establish compliance.

### Outcome measures

- Hysterectomy rates: Age and deprivation index standardised rates; histology, consultant and unit specific rates.

- Proportion of women who have smear tests that undergo a hysterectomy procedure by histology and also by age, deprivation index, hospital and indication for surgery.

- Proportions of those women having a hysterectomy which results in the histology being reported as benign/CIN/malignant.

- Proportion of those having a hysterectomy that are followed up by vault smear tests, with respect to histology group (benign/CIN/malignant), age, deprivation index, hospital, origin of test (primary or secondary care) consultant and indication for surgery.

- Analysis of over/under use of vaginal vault smears with respect to current national guidelines.

## Discussion

Pilot work suggests that too many vault smear tests are probably being undertaken and that this test has poor sensitivity to detect disease in low risk women. These findings fit with some published work but there is a dearth of high quality research in this area.

This research will establish which women are currently having hysterectomy operations and determine when vault smear cytology tests are being done, both in terms of time elapsed post-operatively and histological indication for the test. Thus, compliance with current guidelines will be established and evidence to support the development of future guidelines will be generated.

## Abbreviations

Hospital Episode Statistics = HES. Multi-Centre Research Ethics Committee = MREC. Patient Information Advisory Group = PIAG. Papanicolaou smear test = Pap test. Date of Birth = DOB. National Health Service Cervical Screening Programme = NHSCSP. Cervical Intraepithelial Neoplasia = CIN. Security and Confidentiality Advisory Group of HES = SCAG. NHS Information Authority = NHSIA.

## Competing interests

The author(s) declare that they have no competing interests.

## Authors' contributions

HSL and SW conceived the idea for the study, JM advised during the protocol development. HSL will be undertaking the research with SW and JM providing supervision and statistical expertise. HSL wrote the first draft of this manuscript but all authors have contributed to and approved the final version.

## Pre-publication history

The pre-publication history for this paper can be accessed here:



## References

[B1] Farquhar CM, Steiner CA (2002). Hysterectomy rates in the United States 1990 – 1997. Obstetrics & Gynaecology.

[B2] Hospital Episode Statistics on line data for 2005. http://www.hesonline.nhs.uk/Ease/servlet/ContentServer?siteID=1937&categoryID=204.

[B3] Vessey MP, Villard-Mackintosh L, McPherson K, Coulter A, Yeates D (1992). The epidemiology of hysterectomy: findings in a large cohort study. Br J Obstet Gynaecol.

[B4] Marshall SF, Hardy RJ, Kuh D (2000). Socioeconomic variation in hysterectomy up to age 52: national, population based prospective cohort study. BMJ.

[B5] Gupta, Manyonda I (2006). Hysterectomy for benign gynaecological disease. Current Obstetrics & Gynaecology.

[B6] Maresh MJA, Metcalfe MA, McPherson K, Overton C (2002). The VALUE national hysterectomy study: description of the patients and their surgery. BJOG.

[B7] Edozien L (2005). Hysterectomy for benign conditions. BMJ.

[B8] Pisctilli JT, Bastia LA (1995). Cytologic screening after hysterectomy for benign disease. AmJOG.

[B9] Videlefsky A, Grossl N, Denniston M, Sehgal R (2000). Routine vaginal cuff smear testing in post hysterectomy patients with benign uterine conditions: when is it indicated?. Journal of the American Board of Family Practice.

[B10] Heller DS, Kambham N, Smith D, Cracchiolo B (1999). Recurrence of gynaecologic malignancy at the vaginal vault after hysterectomy. IntJGO.

[B11] Kirkup W, Singer A, Hill AS (1979). Follow-up of women treated for cervical pre-cancer: an argument for a more rational approach. Lancet.

[B12] Gemmell J, Holmes DM, Duncan ID (1990). How frequently need vaginal smears be taken after hysterectomy for cervical intraepithelial neoplasia?. BJOG.

[B13] Stokes-Lampard H, Wilson S, Waddell C, Ryan A, Holder R, Kehoe S (2006). Vaginal vault smears after hysterectomy for reasons other than malignancy: a systematic review of the literature. BJOG.

[B14] Eaker E, Vierkant R, Konitzer K, Remington P (1998). Cervical cancer screening among women with and without hysterectomies. Obstetrics and Gynaecology.

[B15] Bliss P, Trott PA, Blake PR (1997). Cost effectiveness of routine cytological cervical surveillance following treatment for carcinoma of cervix. J Clin Eff.

[B16] Bankhead C, Austoker J, Davey C (2003). Cervical screening results explained: a guide for primary care. CRUK.

[B17] Luesley D, Leeson S, NHSCSP (2004). Colopscopy and Programme Management.

[B18] Stokes-Lampard HJ (2003). The role of vault smears in Primary Care: A questionnaire based survey of Primary Health Care Practitioners. MSc thesis.

[B19] Fox J, Remington P, Layde P, Klein G (1999). The effect of hysterectomy on the risk of an abnormal screening Papanicolaou test result. Am J Obstet Gynaecol.

[B20] NHS connecting for health explanation of NHS number. http://www.connectingforhealth.nhs.uk/systemsandservices/nhsnumber.

[B21] (2004). The English Indices of 2004: Summary (revised) Office of the Deputy Prime Minister. http://www.communities.gov.uk/publications/communities/englishindices.

[B22] (2004). West Midlands Key Health Data 2003. DPHE.

[B23] HES Extract Pack, Hospital Episode Statistics. http://www.dh.gov.uk/PublicationsAndStatistics/Statistics/HospitalEpisodeStatistics/fs/en.

[B24] 2002–03 Annual report of the NHSIA. 2003-IA-1353. NHS Information Authority.

